# Potentialities of multi-b-values diffusion-weighted imaging for predicting efficacy of concurrent chemoradiotherapy in cervical cancer patients

**DOI:** 10.1186/s12880-020-00496-x

**Published:** 2020-08-14

**Authors:** Bing Liu, Wan-Ling Ma, Guang-Wen Zhang, Zhen Sun, Meng-Qi Wei, Wei-Huan Hou, Bing-Xin Hou, Li-Chun Wei, Yi Huan

**Affiliations:** 1 Department of Radiology, Xijing Hospital, Fourth Military Medical University, 127 Changle Western Road, Xi’an, P. R. China 710032; 2 Department of radiology, Longgang District People’s Hospital, Shenzhen, Guangdong P. R. China 518172; 3 Department of Orthopaedics, Xijing Hospital, Fourth Military Medical University, 127 Changle Western Road, Xi’an, P. R. China 710032; 4 Department of Radiation Oncology, Xijing Hospital, Fourth Military Medical University, 127 Changle Western Road, Xi’an, P. R. China 710032

**Keywords:** Cervical cancer, Diffusion magnetic resonance imaging, Concomitant Chemoradiotherapy

## Abstract

**Background:**

To testify whether multi-b-values diffusion-weighted imaging (DWI) can be used to ultra-early predict treatment response of concurrent chemoradiotherapy (CCRT) in cervical cancer patients and to assess the predictive ability of concerning parameters.

**Methods:**

Fifty-three patients with biopsy proved cervical cancer were retrospectively recruited in this study. All patients underwent pelvic multi-b-values DWI before and at the 3rd day during treatment. The apparent diffusion coefficient (ADC), true diffusion coefficient (D_slow_), perfusion-related pseudo-diffusion coefficient (D_fast_), perfusion fraction (f), distributed diffusion coefficient (DDC) and intravoxel diffusion heterogeneity index(α) were generated by mono-exponential, bi-exponential and stretched exponential models. Treatment response was assessed based on Response Evaluation Criteria in Solid Tumors (RECIST v1.1) at 1 month after the completion of whole CCRT. Parameters were compared using independent *t* test or Mann-Whitney *U* test as appropriate. Receiver operating characteristic (ROC) curves was used for statistical evaluations.

**Results:**

ADC-T0 (*p* = 0.02), D_slow_-T0 (*p* <  0.01), DDC-T0 (*p* = 0.03), ADC-T1 (*p* <  0.01), D_slow_-T1 (*p* <  0.01), ΔADC (*p* = 0.04) and Δα (*p* <  0.01) were significant lower in non-CR group patients. ROC analyses showed that ADC-T1 and Δα exhibited high prediction value, with area under the curves of 0.880 and 0.869, respectively.

**Conclusions:**

Multi-b-values DWI can be used as a noninvasive technique to assess and predict treatment response in cervical cancer patients at the 3rd day of CCRT. ADC-T1 and Δα can be used to differentiate good responders from poor responders.

## Background

Diffusion-weighted imaging (DWI) is sensitive to water molecular diffusion within biological tissues. Apparent diffusion coefficient (ADC) derived from mono-exponential model (MEM) is still the most adopted parameter in guiding daily clinical work nowadays. The ADC values may not reflect water diffusion in tissue accurately, because it is also influenced by the microcirculation perfusion in capillaries [1]. Based on bi-exponential model (BEM), multi-b-values DWI might enable to separate the microcirculation perfusion from true diffusion [1, 2]. Stretched exponential model (SEM) offers information on heterogeneity of intravoxel diffusion rates and the distributed diffusion effect, thus providing complementary information of tissue property [3]. MEM, BEM and SEM DWI models have already been applied as imaging biomarker to predict and assess treatment response in rectal cancer, head and neck squamous cell carcinoma, breast cancer, prostate cancer and esophageal squamous cell carcinoma [4–15]. Several reports revealed that MEM, BEM and SEM DWI models could be used in the diagnosis, differentiation and separation of type and grade in cervical cancer (CC) [16, 17]. BEM DWI models were useful for predicting and monitoring the treatment efficacy CC patients [18–20], but results were contradictory. To date, SEM DWI models has not been used in the prediction and assessment of treatment response in CC.

Treatment options differ according to tumor Federation of Gynecology and Obstetrics (FIGO) stage and lymph node status; early-stage disease (IA and IB1) is treated by surgery alone, whereas locally advanced (IB2, IIA2 and IIB to IVA) or lymph node positive diseases is treated with CCRT. It is generally agreed that tumor volume diminish is a favorable indicator of good treatment response [21, 22] and volume reduction is related with local control in CC patients underwent concurrent chemoradiotherapy (CCRT) [23]. Currently researches concerning treatment response prediction mainly focus on parameters change at 1 week and 4 weeks after treatment initiation [19, 24, 25], but no earlier time-points have been evaluated. With the increase of chemoradiotherapy dose, toxicity and adverse side effects aggravate in CC patients during CCRT. Therefore, it is valuable to search an ultra-early time-point to evaluate the treatment response. Tumor molecular changes generally happen earlier than morphological change during CCRT in CC. In order to search an earlier time-point to identify good responders from poor responders, we set the completion of third external beam radiotherapy (EBRT) (at a dose of 6 Gy) as ultra-early monitoring point by using multi-b-values DWI. In order to investigate tumor diffusion property change accurately during CCRT between good and poor responders, mono-exponential, bi-exponential, and stretched exponential DWI models were performed.

The present study aimed to search for a potential early imaging biomarker to predict treatment response of CCRT in CC patients at early stage by using multi-b-values DWI parameters.

## Methods

### Patients

This study was approved by the ethics committee of Institutional Review Board of our hospital, and written informed consents were obtained from all patients before participation. Between Nov 2018 and May 2019, 53 consecutive patients with histologically proven untreated CC scheduled to undergo CCRT treatment were enrolled in this retrospective study. The exclusion criteria were contradictions for MR scanning or CCRT. There was no dropout in our research.

### CCRT treatment

All patients were treated with a combination of EBRT and intracavitary brachytherapy (ICBT). EBRT was delivered to the whole pelvis, with a total dose of 50 Gy (daily dose of 2 Gy, 5 times per week) and accompanied by concurrent chemotherapy: six cycles of weekly cisplatin (40 mg/m^2^) or three cycles of cisplatin (75 mg/m^2^) at 3-week intervals. ICBT was initiated after an EBRT dose of 46–50 Gy. ICBT was delivered once or twice a week in 4–5 fractions, with a fractional dose of 6–7 Gy at point A. The median dose of ICBT was 28 Gy and the median biological effective dose (BED) was 47.8 Gy (range, 23.3–64.7 Gy) to point A.

### MRI protocol

All patients underwent MR examination at two time-points: within 1 week before (T0) and the 3rd day during (T1) CCRT. All MR examinations were performed on a 3.0 T MRI scanner (GE Healthcare 750 Discovery, Milwaukee, Wisconsin, USA) using an 8-channel phase array coil. Routine MRI protocols included sagittal T2WI (repetition time [TR]/echo time [TE]: 4763 /85 ms; slice thickness/spacing: 4 /0.4 mm; field of view [FOV]: 28 cm; number of excitations [NEX]: 4), coronal T2WI (TR/TE: 4171 /85 ms; slice thickness/spacing: 5 / 0.5 mm; FOV: 32 cm; NEX: 4), axial T2WI with fat suppression (TR/TE: 4580 /85 ms; slice thickness/spacing: 4 /1 mm; FOV: 34 cm; NEX: 4), axial T1WI (TR/TE: 601 /minimum ms; slice thickness/spacing: 3 /1 mm; FOV: 32 cm; NEX: 2). Axial multi-b-values DWI with 11 b-values of 0, 10, 20, 40, 80, 150, 200, 400, 800, 1000 and 1200 s/mm^2^ was performed with a single-shot echo-planar sequence (TR/TE: 3883 /59 ms; slice thickness/spacing: 5 /0.5 mm; FOV: 36 cm; matrix, 128 × 160; NEX 1 to 6 with the increasing of b-values, total scan time 6:34 min).

### Treatment response assessment

Treatment response was assessed at 1 month after the completion of the entire CCRT by using convention MR scanning according to the evaluation criteria in solid tumors (RECIST v1.1 [26]) as follows: (1) complete response (CR): no residual tumor showed on the MR images; (2) partial response (PR): the largest diameter of residual tumor was at least 30% less than the original size; (3) progressive disease (PD): there was an at least 20% increase in the longest diameter of tumor compared with the pretreatment size; (4) stable disease (SD): there was neither a decrease sufficient to qualify for PR nor an increase sufficient to qualify for PD. All patients were dichotomized into two groups, CR group and non-CR group. The CR group consisted of patients with CR, while non-CR group consisted of patients with PR, SD and PD.

### Image analysis

Two radiologists with 15 and 2 years’ experience in gynecologic imaging performed post-process and image analysis independently. Readers were blinded to the pathological findings and therapeutic responses. All functional parameters maps were post-processed by using the MADC program on the Advantage Workstation (AW 4.6 version, GE, US). The regions of interest (ROIs) containing all the tumor region and avoiding obvious necrotic areas were manually delineated along the margin of tumor on the three consecutive maximal tumor slices on axial DWI images with b = 1000 s/mm^2^. The mean value of parameters of the three ROIs was used for statistical analysis.

The mono-exponential model was applied to calculate ADC value from all 11 b values by using the following equation [1]:

1 $$ \mathrm{S}/{\mathrm{S}}_0=\exp \left(\hbox{-} \mathrm{b}\cdot \mathrm{ADC}\right) $$

Where S_0_ and S represent the signal intensity obtained with the b = 0 and b > 0 s/mm^2^.

The bi-exponential model, also called intravoxel incoherent motion (IVIM), was applied to calculate D_slow_, D_fast_, and f_p_ values with the following equation [27]:

2 $$ {\mathrm{S}}_{\mathrm{b}}/{\mathrm{S}}_0=\left(1\hbox{-} {\mathrm{f}}_{\mathrm{p}}\right)\cdot \exp \left(\hbox{-} \mathrm{b}\cdot {\mathrm{D}}_{\mathrm{slow}}\right)+{\mathrm{f}}_{\mathrm{p}}\cdot \exp \left(\hbox{-} \mathrm{b}\cdot {\mathrm{D}}_{\mathrm{f}\mathrm{ast}}\right) $$

Where S_b_ represents the mean signal intensity with diffusion gradient b, and S_0_ represents the mean signal intensity at b = 0 s/mm^2^. The f_p_ (perfusion fraction) represents the ratio of water movement within capillaries compared with the total volume of water in a voxel. D_slow_ (pure diffusion coefficient) represents pure molecular diffusion where a physiological perfusion effect is excluded. D_fast_ (pseudo-diffusion coefficient) represents the average blood velocity and mean capillary segment length. Considering that D_fast_ is much greater than D_slow_ with one order of magnitude, the effects of D_fast_ on the signal decay at large b-values (> 200 s/mm^2^) can be ignored.

The stretched exponential model was used to calculate DDC and α by using the following equation [3]:

3 $$ \mathrm{S}/{\mathrm{S}}_0=\exp \left(\hbox{-} {\left(\mathrm{b}\cdot \mathrm{DDC}\right)}^{\upalpha}\right) $$

Where S_0_ and S represents the signal intensity obtained with the b = 0 and b > 0 s/mm^2^. DDC represents the distributed diffusion coefficient reflecting the mean intravoxel diffusion rate, while α represents intravoxel diffusion heterogeneity index corresponding to intravoxel water molecular diffusion heterogeneity with a range from 0 to 1 [28].

### Statistical analysis

All statistical analyses were performed using SPSS (Version 17.0, SPSS Inc., Chicago, IL, USA) and GraphPad Prism 5 (GraphPad Prism Software Inc., San Diego, California, USA). An intra-class correlation coefficient (ICC) was calculated to evaluate interobserver reliability of the measurements. Change of MRI parameters(Δ) was defined as (parameter-T1-parameter-T0)。All quantitative values are presented as the mean ± standard deviation (SD). Clinical characteristics of cervical cancer patients with different treatment outcome was compared using Chi-square test. The Kolmogorov–Smirnov test was conducted to analyze the normal distribution of all metrics. Comparisons between CR group and non-CR group, and between different time-points were performed by using independent t test (D_slow_, DDC and α, which conformed to normal distribution) and Mann–Whitney U test (ADC, D_fast_ and f_p_, which did not conform to normal distribution). Two-tailed *p* values were used and p values less than 0.05 were considered as statistically significant. The area under the curve (AUC) of the receiver operating characteristic (ROC) curves for the significant parameters were calculated and compared. The cut-off values were selected by using the maximized values of the Youden indexes. The values that corresponded to the highest Youden index were chosen as the optimal threshold values.

## Results

### Patients and treatment characteristics

**Fig. 1:**
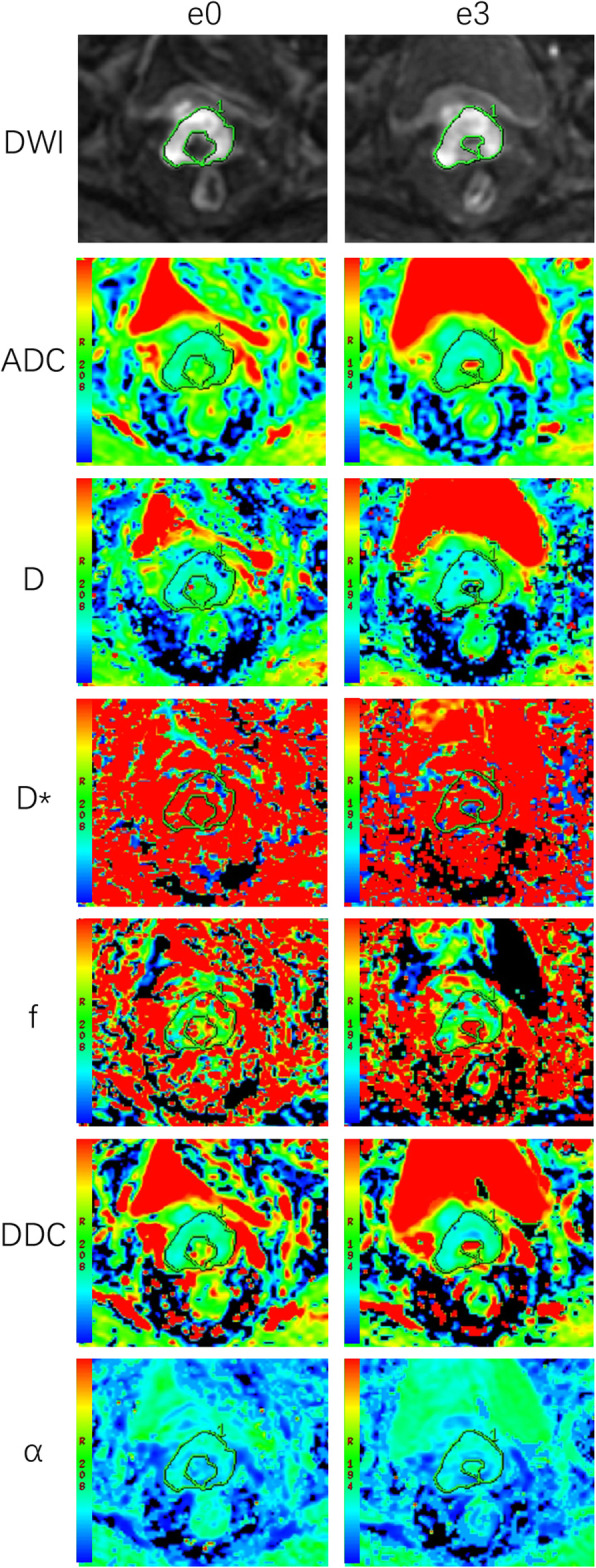
A cervical cancer patient from the complete response group. Images in first line were DWI, ADC, D_slow_, D_fast_, f_p_, DDC and α maps at T0(before CCRT). The ADC, D_slow_, D_fast_, f_p_, DDC and α values were 0.97 × 10^− 3^ mm^2^/s, 0.61 × 10^− 3^ mm^2^/s, 99.02 × 10^− 3^ mm^2^/s, 0.21, 0.92 × 10^− 3^ mm^2^/s and 0.76, respectively. Images in second line were DWI, ADC, D, D*, f, DDC and α at T1 (the 3rd day during CCRT) of the same patient. The ADC, D_slow_, D_fast_, f_p_, DDC and α values were 1.71 × 10^− 3^ mm^2^/s, 1.44 × 10^− 3^ mm^2^/s, 81.82 × 10^− 3^ mm^2^/s, 0.23, 2.53 × 10^− 3^ mm^2^/s and 0.65, respectively

**Fig. 2:**
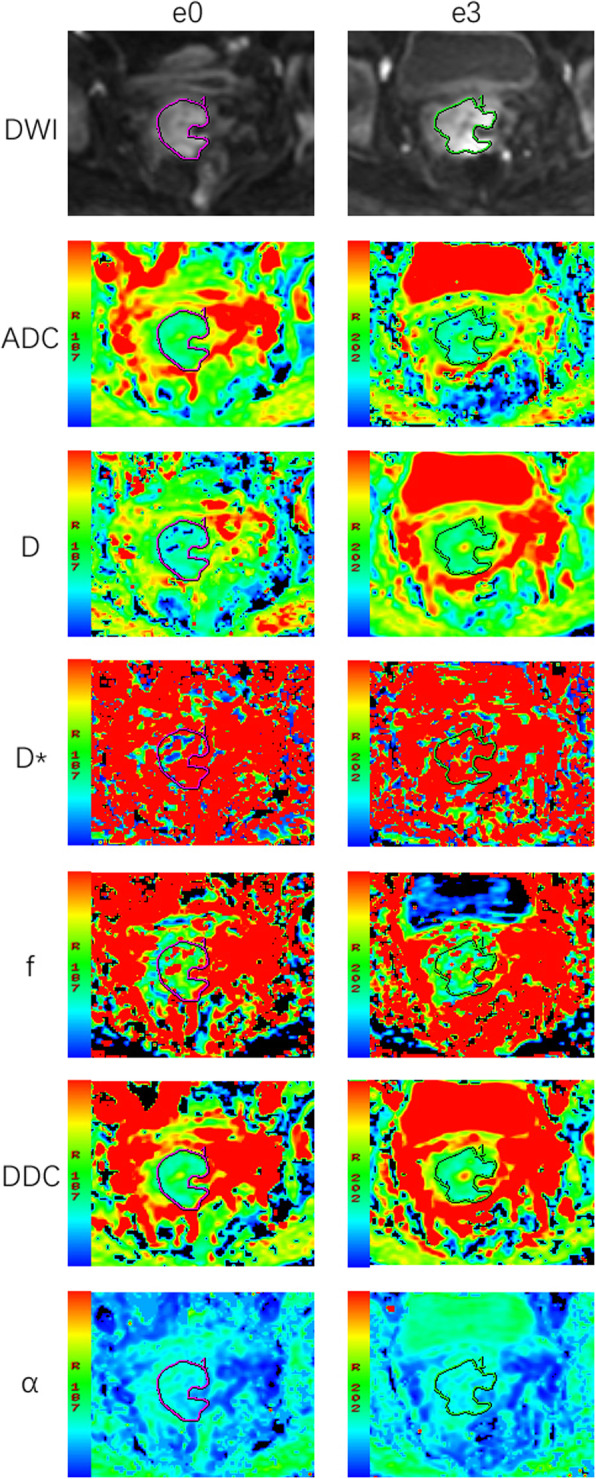
A cervical cancer patient from the non-complete response group. Images in first line were DWI, ADC, D_slow_, D_fast_, f_p_, DDC and α maps at T0 (before CCRT). The ADC, D_slow_, D_fast_, f_p_, DDC and α values were 1.04 × 10^− 3^ mm^2^/s, 0.90 × 10^− 3^ mm^2^/s, 99.02 × 10^− 3^ mm^2^/s, 0.15, 0.33 × 10^− 3^ mm^2^/s and 0.70, respectively. Images in second line were DWI, ADC, D, D*, f, DDC and α at T1 (the 3rd day during CCRT) of the same patient. The ADC, D_slow_, D_fast_, f_p_, DDC and α values were 1.06 × 10^− 3^ mm^2^/s, 0.81 × 10^− 3^ mm^2^/s, 76.42 × 10^− 3^ mm^2^/s, 0.22, 1.06 × 10^− 3^ mm^2^/s and 0.76, respectively

Patients and treatment characteristics were listed in Table 1. The final study cohort included 53 CC patients with FIGO II-IVB disease (mean age: 51.2 years, range 27–67 years). One month after the completion of CCRT, MRI examination showed that 35 of the 53 patients (66.04%) achieved CR and 18 patients (33.96%) achieved incomplete response. No significant differences were observed between patient groups in terms of clinical characteristics. Figures 1 and 2 provided functional parameter maps of CR and non-CR patients before and during CCRT.

**Table 1:** Clinical characteristics between cervical cancer patients with different treatment outcome

Clinical characteristics	CR	non-CR	*t* or *X*^2^	*p*
Number of patients	35	18		
Age (years)	52.4	51.7	0.36^a^	0.72
FIGO stage			0.08^b^	0.78
II	17	8		
III+ IV	18	10		
Histology			1.67^b^	0.20
Squamous cell carcinoma	33	15		
Adenocarcinoma	2	3		
Lymph node status			0.58^b^	0.45
Positive	25	11		
Negative	10	7		

Note: Data are number (%) or mean (range), *FIGO* The International Federation of Gynecology and Obstetrics, *CR* Complete response. ^a^ Comparisons were performed by independent t test. ^b^ Comparisons were performed by Chi-square test

### Interobserver agreement in imaging analysis

The intraclass correlation coefficients (ICCs) of all parameters were ranging between 0.852 to 0.934, which means the measurements of MEM, BEM and SEM derived parameters had good interobserver reproducibility. Details were presented in Table 2.

**Table 2:** Interobserver consistency of DWI derived parameters

	ICC	95% confidence interval
ADC (× 10^− 3^ mm^2^/s)	0.934	0.913–0.958
D_slow_ (× 10^− 3^ mm^2^/s)	0.913	0.891–0.936
D_fast_ (× 10^− 3^ mm^2^/s)	0.852	0.817–0.913
f_p_	0.902	0.880–0.935
DDC (×10^− 3^ mm^2^/s)	0.919	0.905–0.943
α	0.931	0.926–0.957

Note: *ICC* Intraclass correlation coefficient

### Comparison of MRI parameters between CR group and non-CR group

The differences of ADC, D_slow_, D_fast_, f_p_, DDC and α values between patients with different clinical outcome were presented in Table 3. Our results revealed that pretreatment ADC-T0 (0.94 × 10^− 3^ vs 1.08 × 10^− 3^ mm^2^/s, *p* = 0.02), D_slow_-T0 (0.76 × 10^− 3^ vs 0.93 × 10^− 3^ mm^2^/s, *p* <  0.01) and DDC-T0 (1.02 × 10^− 3^ vs 1.20 × 10^− 3^ mm^2^/s, *p* = 0.02) was significantly lower in non-CR group patients. At the 3rd day during CCRT, ADC-T1 (1.26 × 10^− 3^ vs 1.00 × 10^− 3^ mm^2^/s, *p* <  0.01) and D_slow_-T1 (1.07 × 10^− 3^ vs 0.92 × 10^− 3^ mm^2^/s, *p* <  0.01) were significantly higher in CR group patients. Between the two time-points, the changes of ADC (ΔADC: 0.18 × 10^− 3^ vs 0.05 × 10^− 3^ mm^2^/s, *p* = 0.04) and α (Δα: 0.03 vs 0.01, *p* < 0.01) were significantly bigger in CR group patients. No significant differences were found in D_fast_-T0, D_fast_-T1, ΔD_fast_, f_p_-T0, f_p_-T1, Δf_p_, DDC-T1, ΔDDC, α-T0 and α-T1 between the two groups (*p* > 0.05).

**Table 3:** Comparison of DWI derived parameters between patients with different treatment outcomes

	CR	non-CR	*t* /*z*	*p*
ADC-T0 (× 10^− 3^ mm^2^/s)	1.08 ± 0.20	0.94 ± 0.18	189.50^b^	0.02^*^
ADC-T1 (×10^− 3^ mm^2^/s)	1.26 ± 0.16	1.00 ± 0.16	75.50 ^b^	< 0.01^*^
ΔADC (× 10^− 3^ mm^2^/s)	0.18 ± 0.20	0.05 ± 0.17	202.50 ^b^	0.04^*^
D_slow_-T0 (× 10^− 3^ mm^2^/s)	0.93 ± 0.15	0.76 ± 0.13	4.12^a^	< 0.01^*^
D_slow_-T1 (×10^−3^ mm^2^/s)	1.07 ± 0.16	0.92 ± 0.16	3.31 ^a^	< 0.01^*^
ΔD_slow_ (×10^−3^ mm^2^/s)	0.14 ± 0.24	0.17 ± 0.21	0.36 ^a^	0.72
D_fast_-T0 (×10^−3^ mm^2^/s)	71.63 ± 14.82	69.78 ± 15.57	253.50 ^b^	0.25
D_fast_-T1 (×10^−3^ mm^2^/s)	88.59 ± 10.13	82.83 ± 15.32	215.50 ^b^	0.06
ΔD_fast_ (×10^−3^ mm^2^/s)	16.95 ± 20.31	13.06 ± 21.27	284.5 ^0b^	0.57
f_p_-T0	0.26 ± 0.06	0.25 ± 0.06	289.50 ^b^	0.64
f_p_-T1	0.28 ± 0.06	0.28 ± 0.07	308.00 ^b^	0.90
Δf_p_	0.02 ± 0.07	0.03 ± 0.10	286.00 ^b^	0.59
DDC-T0 (×10^−3^ mm^2^/s)	1.20 ± 0.29	1.02 ± 0.27	2.31 ^a^	0.02^*^
DDC-T1 (×10^−3^ mm^2^/s)	1.29 ± 0.28	1.29 ± 0.27	0.07 ^a^	0.94
ΔDDC (×10^−3^ mm^2^/s)	0.08 ± 0.44	0.28 ± 0.31	1.66 ^a^	0.10
α-T0	0.65 ± 0.10	0.66 ± 0.06	0.22 ^a^	0.82
α-T1	0.68 ± 0.10	0.67 ± 0.06	0.41 ^a^	0.68
Δα	0.03 ± 0.02	0.01 ± 0.01	3.59 ^a^	< 0.01^*^

Note: Data are expressed as mean ± SD. * *p* < 0.05

^a^Comparisons were performed by independent t test. ^b^ Comparisons were performed by Mann–Whitney U test

### ROC analysis of MRI parameters

**Fig. 3:**
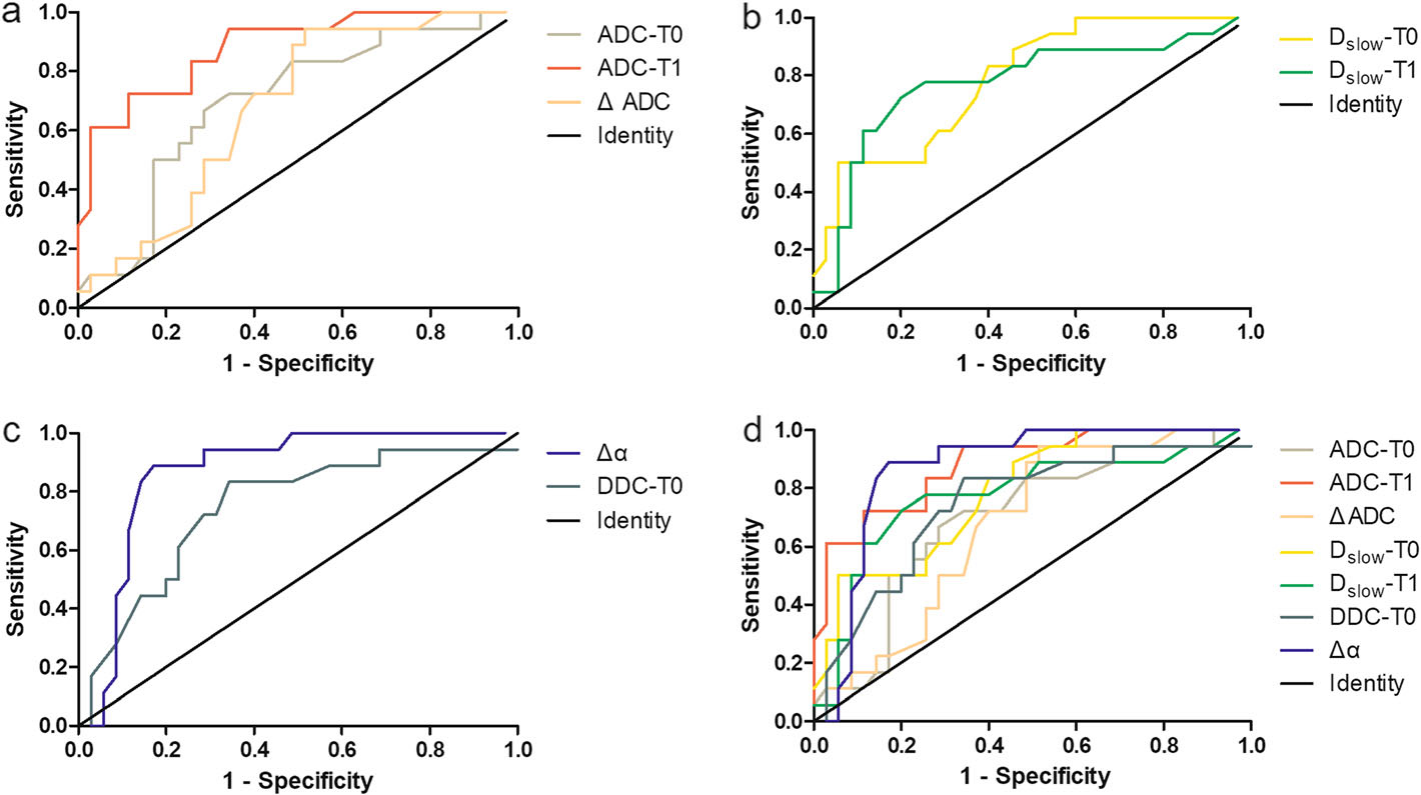
ROC curves of DWI derived parameters in differentiating the good responders from poor responders. **a** The AUCs of MEM derived parameters ADC-T0, ADC-T1 and ΔADC were 0.699, 0.880 and 0.679, respectively. **b** The AUCs of BEM derived parameters D_slow_-T0 and D_slow_-T1 were 0.787 and 0.774. **c** The AUCs of SEM derived parameters Δα and DDC-T0 were 0.869 and 0.745. **d** ADC-TI and Δα showed higher diagnostic accuracy with AUCs above 0.8 among DWI derived parameters. ROC = receiver operating characteristic curves, DWI = diffusion-weighted imaging, AUCs = areas under the curve

The results of ROC analyses of DWI-derived parameters were presented in Table 4 and Fig. 3. The ROC analysis indicated that ADC-T1 showed the highest predictive value, with an AUC of 0.880, closely followed by Δα (AUC = 0.869). The predictive values of ADC-T0, ΔADC, D-T0, D-T1 and DDC-T0 were low, with AUCs below 0.80. By adopting these parameters into treatment response prediction, ADC-T1 showed high predictive sensitivity of 83.78%, specificity of 75.00%, positive predictive value of 88.57% and negative predictive value of 66.67%, while Δα showed high predictive sensitivity of 90.91%, specificity of 75.00%, positive predictive value of 85.71% and negative predictive value of 83.33%.

**Table 4:** Sensitivity, specificity, PPV, and NPV of parameters at optimal cutoff values for differentiate patients with different treatment outcomes

Parameters	AUC (95%CI)	Optimal cutoff value	Sensitivity (%)	Specificity (%)	PPV (%)	NPV (%)
ADC-T0	0.699 (0.551–0.847)	0.995(×10^− 3^ mm^2^/s)	82.14	52.00	65.71	72.22
ADC-T1	0.880 (0.786–0.975)	1.050(×10^− 3^ mm^2^/s)	83.78	75.00	88.57	66.67
ΔADC	0.679 (0.535–0.822)	0.185(×10^−3^ mm^2^/s)	94.12	47.22	45.71	94.44
D_slow_-T0	0.787 (0.663–0.910)	0.760(×10^−3^ mm^2^/s)	78.57	81.82	94.29	50.00
D_slow_-T1	0.774 (0.629–0.919)	0.990(×10^−3^ mm^2^/s)	86.67	60.87	74.29	77.78
DDC-T0	0.745 (0.601–0.890)	1.140(×10^− 3^ mm^2^/s)	88.46	55.56	65.71	83.33
Δα	0.869 (0.768–0.970)	0.022	90.91	75.00	85.71	83.33

Note: *AUC* Area under the curve, *PPV* Positive predictive value, *NPV* Negative predictive value

## Discussion

In present study, we applied multi-b-values DWI derived perfusion and diffusion parameters for ultra-early prediction of treatment response to CCRT in CC patients. The present study revealed the different perfusion and diffusion characteristics between CR and non-CR group patients on the basis of MEM, BEM and SEM DWI models. The results of this study showed that pretreatment diffusion parameters including ADC-T0, D_slow_-T0, and DDC-T0 were significantly higher in CR group patients. Pretreatment CR group patients possess better water diffusion property than non-CR group patients, which may be due to relative loose cellularity or consistent distribution. This resulted in higher sensitivity to treatment regime in CR group patients. Better perfusion of the tumor helps delivery of cytotoxic drugs as well as oxygen during radiation therapy [10], but we didn’t find difference between D_fast_ and *f*_p_, this may be caused by the complexity of microcirculation perfusion.

Moreover, our study demonstrated that the completion of 3rd day can be a feasible time-point to monitor and predict treatment response. Baseline ADC, D_slow_, DDC exhibited diagnostic ability, but diagnostic potency was higher for ADC-T1 and Δα. By monitoring DWI parameters on 3rd day, we can raise accuracy in differentiating patients with different treatment response. Previous studies demonstrated that change of tumor diffusion property can be used as indicator to screen out poor responders in CC patients underwent CCRT [29, 30]. We further advanced the monitoring time-point to the completion of third EBRT, and found that MEM, BEM and SEM DWI derived parameters showed significant difference between good and poor responders. Long before morphological tumor volume reduction, an early increase in water molecular diffusivity may be associated with the reduced tumor cellularity and destructive cell membrane integrity due to apoptosis and necrosis during chemoradiation [10]. By adopting multi-b-values DWI, we can quantitatively measure therapeutic reaction non-invasively in vitro at ultra-early time-point with high accuracy. This may provide supplementary information for prompt and individualized interventions for poor responders to economize medical expenditure and alleviate unnecessary toxicity and complications [31].

Compared with MEM and SEM, BEM derived parameters showed larger fluctuation and poor repeatability. The results in several BEM based studies varied. Wang et al. reported that D_slow_ values were significantly higher for the responders than non-responders before and 3 weeks after neoadjuvant chemotherapy treatment (NACT) initiation in CC patients [18], which was in consistent with our research. Bian *et.al* demonstrated that ADC_min_ and ADC_slow_ of good outcome group were significantly higher than those of poor outcome group. Moreover, at the 7th day of treatment, f and its change rate of good outcome group were significantly higher than those of poor outcome group [20]. Kato et al. reported that no significance was found before and during CRT at a dose of 20 Gy, but the changes of D_slow_, D_fast_ and f_p_ between the two time-points were significantly higher in CR group patients [24]. The above two studies had smaller cohort population and only used 4 and 6 b values to calculated BEM parameters, whereas previous studies used 10 or more b values to calculate BEM parameters in treatment response prediction, therefore this might cause bias in their research. We didn’t find difference in D_fast_ and f_p_ before and during treatment between the two groups. During our research, we found that this might be caused by the poor repeatability and large fluctuation of D_fast_. Che *et.al* also reported this phenomenon [12]. Andreou et al. declared that these could be due to intra tumoral heterogeneity and noise variation [6]. Further research should be conducted in order to illustrate this fluctuation. The f_p_ showed good repeatability but we didn’t find difference between the two groups. The mechanism for an increase in f_p_ is uncertain, but may reflect vascular normalization within tumors [13].

Unlike BEM, SEM was reported to show high precision and excellent repeatability, which was an important consideration when evaluating diffusion models for treatment response prediction [13, 14, 31]. The parameters obtained from SEM were highly repeatable, therefore they might be robust and could be employed as reliable quantitative tools. Our results also support this idea. SEM derived parameters have been used to assess and predict treatment response in brain, breast, rectal, prostate tumors [11, 14, 32, 33]. A very strong positive relationship between ADC, D_slow_ and DDC was found [13, 34]. This may indicate that they are sensitive to the same tissue characteristics and provide similar information. CR group CC patients with the higher DDC-T0 might be more sensitive to drugs within the microenvironment. Tumors with higher cellular and glandular pleomorphism tend to have higher level of intravoxel diffusion heterogeneity thus a lower α [14]. Zhu et al. reported that Δα was higher in patients achieved pathological complete response in locally advanced rectal cancer patients [5], which was consistent with our result. Several studies have shown that high-grade or high-stage tumors exhibited lower α values [29, 33], thereby the increase of α values could be interpreted as the tumor is less aggressive and more sensitive to treatment. Zhang *et.al* also compared value of MEM, BEM and SEM models in treatment response prediction of CC patients. They found that ADC, D_slow_ and DDC was lower in responders than in non-responders groups, and α was higher in responders group than in non-responders group [35], which was inconsistent with our result. This might be caused by the difference in observation endpoint. Zhang *et.al* defined responders as CR or PR patients, and CR was defined as who appeared as CR at anytime during 12 months, while we determined treatment outcome at 1 month after the completion of CCRT. They depicted that higher DDC represented more necrosis and poorer oxygenation, which resulted in extended radiotherapy resistance, thus DDC was higher in non-responder group. But we considered that since DDC was the continuous distribution of ADC within voxel, higher DDC represented better water diffusion property resulting in better radio- and chemo- sensitivity. So higher DDC was observed in patient with better treatment response.

There were several limitations in this study. First, the regions of interest were selected in the maximal solid parts of the tumors instead of the entire tumors, which might lead to selection bias owing to histological heterogeneity of tumors. Second, the follow-up time was relatively short and longer follow-ups needed for further confirmation of our results. Third, more monitoring time-points should be set in order to observe the dynamic changes of parameters as some parameters fluctuate during treatment. Our results were preliminary conclusion and further investigation was going to be proceeded.

## Conclusion

The 3rd day may be a critical ultra-early time-point to assess and predict treatment response. Multi-b-values DWI derived parameters ADC-T1 and Δα have great potential for ultra-early prediction of treatment response of CCRT in CC patients.

## Data Availability

The datasets analyzed in this study are available from the corresponding author on request.

## Abbreviations

α: Intravoxel diffusion heterogeneity index
ADC: Apparent diffusion coefficient
AUC: Area under the curve
BED: Biological effective dose
BEM: Bi-exponential model
CC: Cervical cancer
CCRT: Concurrent chemoradiotherapy
CR: Complete response
DDC: Distributed diffusion coefficient
D_fast_: Pseudo-diffusion coefficient
D_slow_: Pure diffusion coefficient
DWI: Diffusion-weighted imaging
EBRT: External beam radiotherapy
FIGO: Federation of Gynecology and Obstetrics
f_p_: Perfusion fraction
ICBT: Intracavitary brachytherapy
ICC: Intra-class correlation coefficient
IVIM: Intravoxel incoherent motion
MEM: Mono-exponential model
NACT: Neoadjuvant chemotherapy treatment
NPV: Negative predictive value
PPV: Positive predictive value
ROC: Receiver operating characteristic curves
SEM: Stretched exponential model
